# Safety of “hot” and “cold” site admissions within a high‐volume urology department in the United Kingdom at the peak of the COVID‐19 pandemic

**DOI:** 10.1002/bco2.56

**Published:** 2021-01-21

**Authors:** Luke Stroman, Beth Russell, Pinky Kotecha, Anastasia Kantartzi, Luis Ribeiro, Bethany Jackson, Vugar Ismaylov, Adeoye Oluwakanyinsola Debo‐Aina, Findlay MacAskill, Francesca Kum, Meghana Kulkarni, Raveen Sandher, Anna Walsh, Ella Doerge, Katherine Guest, Yamini Kailash, Nick Simson, Cassandra McDonald, Elsie Mensah, Li June Tay, Ramandeep Chalokia, Sharon Clovis, Elizabeth Eversden, Jane Cossins, Jonah Rusere, Grace Zisengwe, Louisa Fleure, Leslie Cooper, Kathryn Chatterton, Amelia Barber, Catherine Roberts, Thomasia Azavedo, Jeffrey Ritualo, Harold Omana, Liza Mills, Lily Studd, Oussama El Hage, Rajesh Nair, Sachin Malde, Arun Sahai, Archana Fernando, Claire Taylor, Benjamin Challacombe, Ramesh Thurairaja, Rick Popert, Jonathon Olsburgh, Paul Cathcart, Christian Brown, Marios Hadjipavlou, Ella Di Benedetto, Matthew Bultitude, Jonathon Glass, Tet Yap, Rhana Zakri, Majed Shabbir, Susan Willis, Kay Thomas, Tim O’Brien, Muhammad Shamim Khan, Prokar Dasgupta

**Affiliations:** ^1^ Department of Urology Guy’s Hospital Guys’ & St Thomas’ NHS Foundation Trust London UK; ^2^ Department of Translational Oncology and Urology Research King’s College London London UK; ^3^ Faculty of Life Sciences and Medicine King’s College London London UK; ^4^ Department of Cancer Imaging School of Biomedical Engineering and Imaging Sciences King’s College London London UK

## Abstract

**Objectives:**

To determine the safety of urological admissions and procedures during the height of the COVID‐19 pandemic using “hot” and “cold” sites. The secondary objective is to determine risk factors of contracting COVID‐19 within our cohort.

**Patients and methods:**

A retrospective cohort study of all consecutive patients admitted from March 1 to May 31, 2020 at a high‐volume tertiary urology department in London, United Kingdom. Elective surgery was carried out at a “cold” site requiring a negative COVID‐19 swab 72‐hours prior to admission and patients were required to self‐isolate for 14‐days preoperatively, while all acute admissions were admitted to the “hot” site.

Complications related to COVID‐19 were presented as percentages. Risk factors for developing COVID‐19 infection were determined using multivariate logistic regression analysis.

**Results:**

A total of 611 patients, 451 (73.8%) male and 160 (26.2%) female, with a median age of 57 (interquartile range 44‐70) were admitted under the urology team; 101 (16.5%) on the “cold” site and 510 (83.5%) on the “hot” site. Procedures were performed in 495 patients of which eight (1.6%) contracted COVID‐19 postoperatively with one (0.2%) postoperative mortality due to COVID‐19. Overall, COVID‐19 was detected in 20 (3.3%) patients with two (0.3%) deaths. Length of stay was associated with contracting COVID‐19 in our cohort (OR 1.25, 95% CI 1.13‐1.39).

**Conclusions:**

Continuation of urological procedures using “hot” and “cold” sites throughout the COVID‐19 pandemic was safe practice, although the risk of COVID‐19 remained and is underlined by a postoperative mortality.

## 1 INTRODUCTION

### 1.1 Background

The spread of COVID‐19, the respiratory disease caused by the virus Severe Acute Respiratory Syndrome Coronavirus 2 (SARS‐CoV‐2) was first reported in the United Kingdom in the week commencing January 27, 2020 and was declared a pandemic by the World Health Organization on March 11, 2020.^1^, ^2^ By March 17, 2020, the British Government postponed all nonurgent surgical operations to reduce spread and reallocate resources to target the disease.^3^ The first peak of the COVID‐19 pandemic in the United Kingdom (UK) ranged from April 1 (6199 new cases) to May 1, 2020 (6201 new cases, the highest recorded date).^4^ To date of publication, the United Kingdom had the 15th greatest number of confirmed cases of COVID‐19 worldwide, with the United States, India, and Brazil having the highest, respectively.^5^ The impact of COVID‐19 on urological services is likely to be significant throughout the world; a worldwide survey of 1004 urological clinicians found 28% of clinics, 30% of outpatient procedures, and 31% of urological surgeries had a delays of greater than 8 weeks. Half of clinicians thought that postponement of urological services would affect treatment and survival.^6^

Patients having surgical operations may be at increased risk of contracting COVID‐19 due to the aerosol generated during anesthesia and the procedure itself, which may mobilize pathogens as well as the immunosuppressive response to surgery.^7^ Postoperative pain limiting respiration and immobility can also make patients more susceptible to respiratory infections. Pulmonary complications related to COVID‐19 reported by the COVIDSurg collaborative, an international multicenter observational study found postoperative respiratory complications occurred in half of patients that had contracted COVID‐19 peri‐operatively (from 7 days preoperatively to 30 days postoperatively) and found 30‐day mortality to be 23.8%.^8^ Given these outcomes it is imperative to prevent peri‐operative contraction of the virus and our center has developed “hot” sites for acute admissions and “cold” sites for elective admissions requiring patients to self‐isolate for a minimum of 14 days and produce a negative COVID‐19 polymerase chain reaction (PCR) swab prior to procedure.

The department described in this study is within a high‐volume tertiary referral center. Guy’s & St Thomas’ NHS Trust was at the center of the pandemic, there were over 1400 cases of COVID‐19 diagnosed and over 330 ventilated in intensive care throughout the pandemic, which is the most in the United Kingdom. Up to 2000 members of staff and 20% of the workforce took sick leave either unwell as a result of the virus or isolating during the period reviewed in this study. The current study helps to inform management in other centers with high incidence of COVID‐19 in the community.

### 1.2 Objectives

The primary objective was to determine the safety of the continuation of urological admissions and procedures through the height of the COVID‐19 pandemic using “hot” and “cold” surgical sites. The primary outcomes were postoperative COVID‐19 infection and mortality related to COVID‐19. The secondary objective was to determine risk factors of contracting COVID‐19 within our cohort to determine which patients are more at risk and assist further preventative strategies.

## 2 PATIENTS AND METHODS

### 2.1 Study design

An observational cohort study of all consecutive patients admitted at the height of the COVID‐19 pandemic from March 1, 2020 to May 31, 2020 across both “hot” acute sites and “cold” elective sites was performed. Peri‐operative infection, complications, and mortality related to COVID‐19 were reported to gauge the safety of the continuation of urological procedures throughout the pandemic. Risk factors for developing COVID‐19 infection within our cohort were determined using multivariate logistic regression analysis. Data are presented in line with the Strengthening the Reporting of Observational Studies (STROBE) statement for cohort studies.^9^

### 2.2 Setting

A single urology department of a tertiary care referral center in London, United Kingdom. Patients managed at the “cold” site were “COVID‐19 protected,” requiring patients to self‐isolate for a minimum of 14 days and produce a negative Roche COVID‐19 polymerase chain reaction (PCR) antigen swab within 72 hours prior to the procedure.^10^ “Hot” site admissions were “risk managed” and included all patients admitted acutely throughout the 3 months of the study, as well as all elective admissions prior to “cold” site opening on March 30, 2020. All patients admitted throughout the study period to the “hot” site were screened for respiratory tract symptoms and temperature on arrival and all patients operated on the “hot” site were screened for COVID‐19 with PCR swab preoperatively. As the local guidance changed throughout the study time all patients from April 1 onward were screened with PCR swab on admission. If patients were diagnosed with COVID‐19 they were managed in isolation or in wards with only COVID‐19 positive patients. All COVID‐19 negative or not suspected patients were managed on wards without isolation but with personal protective equipment used. Interventional radiology procedures and lithotripsy were available on the “hot” site only while intensive care was available on both sites.

On both sites Public Health England personal protective equipment (PPE) guidance was followed; for all inpatient care fluid repellent surgical masks, apron, gloves, and eye protection and for aerosol generating procedures filtering facepiece masks (FFP3), fluid repellent gown, gloves, and eye protection were worn by health‐care professionals.^11^ Elective admissions for extracorporeal shock wave lithotripsy remained on the “hot” site throughout with the same isolation precautions required prior to treatment. Laminar flow theaters were used for anesthetizing and operating on the “cold” site for all cases. On the “hot” site laminar flow theaters were not routinely used unless the patient was determined to be COVID‐19 positive preoperatively. Consultant led‐anesthetic care was available on both sites for all surgical elective operations despite a number of the anesthetic team being redeployed to intensive care or COVID‐19‐specific resuscitation teams.

To improve staff safety training was made available to all medical and nursing staff on correct use of PPE, method of donning and doffing, and use of PPE in resuscitation situations. Any members of staff with confirmed or symptoms of COVID‐19 were required to isolate for 14 days. Routine antibody testing was not made available for staff members until July 2020, after the period of the study.

### 2.3 Participants

All consecutive patients admitted under the care of the urology team. Patients were identified using a prospective consecutive data set of admissions and surgical bookings.

### 2.4 Variables

Variables recorded were age, gender, ethnicity, COVID‐19 positive (yes/no), complications related to COVID‐19 (if positive), respiratory support required for COVID‐19 (if positive), mortality (yes/no), hypertension (>140/90 mmHg), Charlson Comorbidity Index, operation performed, and length of stay (in days).

### 2.5 Data sources

Data were retrospectively collected using a prospective consecutive data set of admissions using trust‐wide electronic patient records and morbidity and mortality. Data were gathered using electronic patient records 30 days from the date of admission. Data were gathered from surrounding hospitals when patients were reported to have presented elsewhere.

### 2.6 Bias

There was a risk of underreporting due patients presenting in other trusts with COVID‐19 infection or subclinical COVID‐19 infection in the community.

### 2.7 Statistical methods

Complications related to COVID‐19 were presented as percentages of the overall cohort and number of patients that underwent surgical procedures. Risk factors for developing COVID‐19 infection within our cohort were determined using multivariate logistic regression analysis. Gender, ethnicity hypertension, operation (yes/no), and COVID‐19 status were analyzed as binary variables, age (≤70 or >70), and Charlson Comorbidity Index as dichotomous variables and length of stay as a continuous variable.

### 2.8 Ethics

This service evaluation/audit was granted institutional approval by the Guy’s and St Thomas’ Hospital and the requirement for consent for data use that was anonymized before analysis was waived.

## 3 RESULTS

### 3.1 Participants and descriptive data

A total of 611 patients, 451 (73.8%) male and 160 (26.2%) female with a median age of 57 (interquartile range 44‐70) were admitted under the urology team (Table 1). Of these, 101 (16.5%) were admitted on the “cold” site and 510 (83.5%) on the “hot” site (Table 1). Surgical procedures were performed in 495 (81%) (Table 2).

**TABLE 1:** Patient demographics

Demographic	“Hot” site	“Cold” site	Overall
Total patients	501 (83%)	101 (17%)	611 (100%)
Operative procedure (%)	395 (79%)	101 (100%)	496 (81%)
Mean age (Interquartile range)	55 (42‐69)	59 (48‐73)	57 (44‐70)
Sex (Male)	374 (75%)	68 (67%)	451 (74%)
Sex (Female)	127 (25%)	33 (33%)	160 (26%)
Median Charlson comorbidity score (range)	2 (0‐10)	3 (0‐13)	3 (0‐13)
Hypertension >140/90 mmHg (%)	122 (24%)	43 (43%)	165 (27%)
Ethnicity‐White (%)	221 (44%)	44 (44%)	265 (43%)
Ethnicity‐Black or Afro‐Caribbean (%)	52 (10%)	6 (6%)	58 (9%)
Ethnicity‐Asian (%)	15 (3%)	0 (0%)	15 (3%)
Ethnicity‐other (%)	23 (5%)	5 (5%)	28 (5%)
Ethnicity‐not specified (%)	199 (40%)	46 (46%)	245 (40%)

**TABLE 2:** All procedures completed

Procedure	Total number	Number at “cold” site	Number at “hot” site	Age (Median)	Gender (Male)	Afro‐Caribbean, Black or Asian	Cancer	Hypertension	Charleston comorbidity index (median)	Length of stay (Median)	Postoperative COVID‐19	Mortality
Nephrectomy/Partial nephrectomy	33	18	15	61	21	2	30	15	4	2	2	0
Cystectomy	8	2	6	73	5	0	8	4	6	10	1	0
Transurethral resection of bladder tumor	25	10	15	73	21	2	25	11	5.5	2	1	1
Robotic assisted radical prostatectomy	18	4	14	59	18	6	18	5	3	2	0	0
Brachytherapy	4	0	4	66	4	0	4	0	4	0	0	0
Ureteroscopy +/− stone fragmentation	90	36	54	55	49	12	4	21	2	1	2	0
Extracorporeal shock wave lithotripsy	82	0	82	47	67	9	0	7	1	0	0	0
Percutaneous nephrolithotomy	2	0	2	53	1	0	0	0	1	6.5	0	0
Cystoscopic insertion of ureteric stent	52	15	37	60	26	12	14	17	2.5	1	0	0
Transurethral resection of prostate/bladder outflow surgery	18	4	14	74.5	18	4	5	11	3.5	2	1	0
Andrological/inguinoscrotal surgery	50	4	46	38.5	50	4	5	10	0	0	0	0
Transperineal prostate biopsy	30	4	26	61	30	8	20	7	3.5	0	0	0
Other cystoscopic procedures	61	3	58	54	40	13	18	16	1	1	1	0
Other robotic/reconstructive procedures	13	4	9	64	6	3	7	4	3	2	0	0
Nephrostomy insertion/IR procedures	10	0	10	70	6	0	5	1	6.5	4	0	0
**All procedures**	**496**	**104**	**392**	**61**	**362**	**75**	**163**	**129**	**3**	**1.5**	**8**	**1**

### 3.2 Primary objective

Overall, COVID‐19 was detected in 20 (3.3%) patients with two (0.3%) mortalities. Of the 495 patients that underwent surgical procedures, eight (1.6%) contracted COVID‐19 postoperatively with one (0.2%) postoperative mortality due to COVID‐19. Of the 20 COVID‐19 positive patients 17 were at the “hot” site and 3 at the “cold” site, two of which were detected preoperatively and one postoperatively.

Supplemental oxygen and antibiotics were required in four patients that developed COVID‐19 while another required additional methylprednisolone and awake proning but avoided intensive care (Table 3). There were two mortalities in patients that contracted COVID‐19; one postoperative mortality following transurethral resection of bladder tumor at the “cold” site with negative preoperative COVID‐19 swab that presented to a local hospital in respiratory failure 14 days following surgery and one patient with palliative metastatic bladder cancer and respiratory failure due to COVID‐19 that did not have an operation.

**TABLE 3:** Management required for COVID positive patients

	All patients (%)	Postoperative patients (%)
No treatment required	13 (65%)	4 (50%)
Supplementary oxygen and antibiotics	4 (20%)	3 (37.5%)
Supplementary oxygen, antibiotics, and steroids	1 (5%)	0 (0%)
Ventilatory support	0 (0%)	0 (0%)
Mortality	2 (10%)	1 (12.5%)
Total	20	8

### 3.3 Secondary objectives

On multivariate analysis, length of stay was the associated with contracting COVID‐19 in our cohort (OR 1.25, 95% CI 1.13‐1.39) (Table 4). Patients with higher Charlson Comorbidity Index of 3 or above also looked to be more at risk (OR 15.24, 95% CI 2.00‐115.77) pointing to comorbidity as a risk factor. Patients having surgical procedures were not at higher risk of contracting COVID‐19 compared to those that did not (OR 1.1, 95% CI 0.31‐3.83).

**TABLE 4:** Risk factors of contracting COVID‐19 following multivariate analysis

Variable	No. of COVID events	OR	95% CI	P for Trend (for continuous variables only)
Age				
≤70	8	1.00	Ref.	
>70	12	2.20	(0.54‐8.99)	
Gender				
Female	4	1.00	Ref.	
Male	16	1.18	(0.38‐3.65)	
Black or Asian Ethnicity				
No	15	1.00	Ref.	
Yes	5	1.83	(0.57‐5.86)	
Cancer				
No	10	1.00	Ref.	
Yes	10	1.13	(0.44‐2.91)	
Hypertension				
No	13	1.00	Ref.	
Yes	7	0.70	(0.26‐1.84)	
Charlson comorbidity index				
0 or 1	1	1.00	Ref.	
2	3	12.15	(1.24‐118.93)	
3+	16	15.24	(2.00‐115.77)	
Operation/procedure				
No	4	1.00	Ref.	
Yes	16	1.10	(0.31‐3.83)	
Length of stay (cont.)	N/A	1.25	(1.13‐1.39)	<0.000
Site of admission				
“Hot” site	17	1.00	Ref.	
“Cold” site	3	0.86	(0.25‐3.01)	

Note

OR—Odds ratio; 95% CI‐95% confidence interval. All odds ratios (OR) were adjusted for minimal variables.

## 4 DISCUSSION

In our cohort the risk of contracting COVID‐19 remained low (3.3%) throughout the peak of the COVID‐19 pandemic in the United Kingdom over both “hot” and “cold” sites. Given that the hospital was at center of the pandemic in London in a severely affected area, this model was a relatively safe method of continuing to deliver urological care. One challenge going forward will be dealing with increased waiting lists and delivering safe care on a larger scale. There was a significant reduction in the elective service during the time of the study; the described department typically has between 600 and 1000 inpatient episodes monthly, in contrast to the 200 per month described in this series. This is a worldwide phenomenon with up to 2.8 million surgeries worldwide expected to be delayed canceled as a result of COVID‐19, making it vital to find a safe method of carrying out operations following the pandemic.^12^ The “cold” site model has been safely described elsewhere, and reports from another “cold” site in London have reported a 2% rate of postoperative COVID‐19 infection, with only 0.2% of their cohort reporting Clavien‐Dindo grade 3 complications related to COVID‐19.^13^ While the “cold” site was not associated with a reduction in COVID‐19 infection when compared to the “hot” site (OR 0.86, 95% CI 0.25‐3.01) it did allow the center described to continue complex important operations safely at the peak of the pandemic. The current study supports the practice of “cold” sites in areas still suffering from high rates of COVID‐19.

Postoperative mortality related to COVID in our cohort was 12.5% (1/8) which is lower than the figure of 20.5% reported in China and 23.8% described by the worldwide COVIDSurg collaborative.^8^, ^14^ Interestingly, apart from the reported mortality there were no Clavien‐Dindo 3 or above complications in the current series related to COVID‐19 although 37.5% (3/8) of postoperative patients required supplementary oxygen and antibiotics. Over the whole cohort there were no patients from our department that required ventilatory or intensive care support apart from the two mortalities. The two patients had significant comorbidities, with Charlson Comorbidity Index scores of 7 and 8, respectively. Multivariate analysis demonstrated that those with greater comorbidity were at a higher risk of contracting COVID‐19, so surgery in these patients must be taken with greater planning and caution in areas with high COVID‐19 prevalence.

An interesting finding of the current study was the association of length of stay with increased COVID‐19 infection. It is unclear whether the patients requiring longer admissions are more at risk, pointing to hospital‐related transmission or if COVID‐19 infection led to a prolonged admission. However, as no patients required intensive care and only five required supplementary oxygen and antibiotics, it is unlikely that the majority of patients with COVID‐19 had an extended stay due to the virus. The three patients with the longest stay with COVID‐19 did not require oxygen and were likely to have been infected while in hospital. This suggests that early discharge where clinically appropriate may aid the reduction in hospital‐related COVID‐19 transmission. In practice surgical departments have attempted to do this and admissions have been reported to have a reduced length of stay during lockdown when compared to previously.^15^ Risk factors for a more severe course of COVID‐19 have been described and include age 65 and older, living in a nursing home or long‐term care facility as well as chronic lung disease.^16^ The rationale for surgery in these patients is particularly important, in the described department all “cold” site surgeries were discussed at a multidisciplinary team meeting and prioritized according to The Royal of College of Surgeons guidance and added to waiting list or deferred according to clinical need.^17^ Surgery in such patients must be taken with extra precautions with patients and the family informed of higher risk of mortality. In the “hot” site decisions to proceed to surgery were made on a case by case basis by the admitting team in conjunction with the peri‐operative medicine team taking the risk of COVID‐19 infection into consideration. While operations for life‐threatening conditions or sepsis were prioritized, important benign conditions were carried out for symptoms, such as ureteroscopies or extracorporeal shock wave lithotripsies for kidney or ureteric stones.

Benefits of “cold” and “hot” site work included the reduced cross‐contamination of COVID‐19 infection from the acute site. Global guidance for surgical care during the COVID‐19 pandemic has suggested patients should be cared for by COVID‐19‐specific surgical teams if possible, rather than those who are also seeing uninfected patients which was followed in our site.^18^ To reduce cross‐contamination, members of the surgical team were not permitted to visit the “cold” site on the same day as the “hot” site although this did not come into effect until a few weeks into the opening of the “cold” site as the “cold” site evolved. Across both sites appropriate PPE guidance was followed in inpatient and outpatient areas according to Public Health England Guidance.^11^ This has been a successful model in our center where two adjacent separate sites were available, however, not all centers have this facility and separation may have to occur within a single site. Appropriate staffing was maintained in the hospital with reallocation of resources, junior doctors from urology were redeployed to support the intensive care department while a reduced junior doctor and advanced nurse practitioner rotation supported the wards of the “hot” site. Personal protective equipment was available throughout and there were sufficient resources to keep to the public heath guidance.^11^ While continuing urological care was safe in the described center, it may not be in centers with more limited human resources, PPE, or access to COVID‐19 PCR testing.

One limitation of this study the possibility of underreporting of postoperative COVID‐19 infection as a result of patients presenting at other hospitals or subclinical infection in the community without presenting to hospital. Asymptomatic shedding of the virus can occur and may account for up to 60% of cases.^16^ To limit this, the authors contacted surrounding hospitals where patients or doctors reported presentations in other sites, as is the case with the postoperative mortality reported. The observational nature of this study meant that the authors did not request patients to have routine postoperative COVID‐19 PCR swabs which may have increased the number of postoperative infections detected. On the contrary, postoperative COVID‐19 infection is not necessarily causative from the admission as COVID‐19 could have been obtained in the community in patients discharged from care. In addition, the “cold” site was not set up at the beginning of the time described and was a response to the pandemic, coming into effect on the March 30, 2020. However, the authors decided to include all patients in March 2020 as this gives a more rounded picture of the height of the pandemic. Prioritization also changed throughout the study, as cases in early March were carried out as previously planned, accounting for some nonurgent benign conditions and low‐risk cancer treatments such as inguinoscrotal surgery and brachytherapy.

It is important to note that not all patients at the “hot” site were tested for COVID‐19 on admission although all “cold” site patients were. Routine testing on the “hot” site changed through the time of the study, initially only symptomatic patients were being tested, while later all emergency admissions were tested. Due to the collection of information using electronic records, ethnicity was not recorded in all patients with 40% not specified on records, meaning conclusions about ethnicity based on this data set were limited. This is an important cohort as black and Asian patients have been associated with worse outcomes when compared to white patients.^19^, ^20^ Due to the low number of mortalities and serious complications related to COVID‐19, we have been unable to make conclusions of whether risk factors are predictors of morbidity or mortality. While this is a single center study, our unit is a high‐volume tertiary urology and oncology center and outcomes here may be able to guide other centers considering “cold” and “hot” site models.

## 5 CONCLUSIONS

Continuation of urological procedures using “hot” and “cold” sites throughout the COVID‐19 pandemic was reasonably safe and enabled patients undergo important procedures, although the risk of COVID‐19 remained and is underlined by a postoperative mortality. We identified risk factors that could limit transmission, notably reducing length of stay.

## CONFLICT OF INTEREST

The Authors do not declare any conflict of interest. Funding has been provided the department of Life Sciences and Medicine, King’s College London.
